# Bioactive Scaffold Fabricated by 3D Printing for Enhancing Osteoporotic Bone Regeneration

**DOI:** 10.3390/bioengineering9100525

**Published:** 2022-10-05

**Authors:** Xiaoting Zhang, Xinluan Wang, Yuk-wai Lee, Lu Feng, Bin Wang, Qi Pan, Xiangbo Meng, Huijuan Cao, Linlong Li, Haixing Wang, Shanshan Bai, Lingchi Kong, Dick Ho Kiu Chow, Ling Qin, Liao Cui, Sien Lin, Gang Li

**Affiliations:** 1 Musculoskeletal Research Laboratory, Department of Orthopaedics & Traumatology, The Chinese University of Hong Kong, Prince of Wales Hospital, Hong Kong, China; 2 Stem Cells and Regenerative Medicine Laboratory, Li Ka Shing Institute of Health Sciences, The Chinese University of Hong Kong, Prince of Wales Hospital, Hong Kong, China; 3 Shenzhen Institute of Advanced Technology, Chinese Academy of Sciences, Shenzhen 518055, China; 4 SH Ho Scoliosis Research Laboratory, Department of Orthopaedics and Traumatology, The Chinese University of Hong Kong, Hong Kong, China; 5 Joint Scoliosis Research Center of the Chinese University of Hong Kong and Nanjing University, The Chinese University of Hong Kong, Hong Kong, China; 6 Li Ka Shing Institute of Health Sciences, The Chinese University of Hong Kong, Hong Kong, China; 7 School of Pharmacy and Guangdong Key Laboratory for Research and Development of Natural Drugs, Guangdong Medical University, Zhanjiang 524023, China

**Keywords:** osteoporotic bone regeneration, PLGA/TCP, icaritin, secretome, additive effect, focal adhesion signalling

## Abstract

We develop a poly (lactic-co-glycolic acid)/β-calcium phosphate (PLGA/TCP)-based scaffold through a three-dimensional (3D) printing technique incorporating icaritin (ICT), a unique phytomolecule, and secretome derived from human fetal mesenchymal stem cells (HFS), to provide mechanical support and biological cues for stimulating bone defect healing. With the sustained release of ICT and HFS from the composite scaffold, the cell-free scaffold efficiently facilitates the migration of MSCs and promotes bone regeneration at the femoral defect site in the ovariectomy (OVX)-induced osteoporotic rat model. Furthermore, mechanism study results indicate that the combination of ICT and HFS additively activates the Integrin–FAK (focal adhesion kinase)–ERK1/2 (extracellular signal-regulated kinase 1/2)–Runx2 (Runt-related transcription factor 2) axis, which could be linked to the beneficial recruitment of MSCs to the implant and subsequent osteogenesis enhancement. Collectively, the PLGA/TCP/ICT/HFS (P/T/I/S) bioactive scaffold is a promising biomaterial for repairing osteoporotic bone defects, which may have immense implications for their translation to clinical practice.

## 1. Introduction

Osteoporosis, characterised by bone loss and microarchitectural dysfunction of bone tissue, is a systemic skeletal disease that primarily affects the elderly, especially postmenopausal women [1,2]. As estrogen levels decline during menopause, there is an imbalance between bone resorption and bone formation, which leads to a decrease in bone mass and strength, and an increase in osteoporotic fracture risks [3,4]. Osteoporotic bone regeneration is impaired due to complicated mechanisms. An important feature of the osteoporotic condition is the absence of osteoblast progenitors, which results from the loss of the proliferation, recruitment, and differentiation ability of MSCs [5]. Therefore, it is imperative to seek therapeutic interventions for osteoporotic fracture treatment by activating MSCs function and enhancing the bone regeneration capacity.

The treatment of osteoporotic fractures is a challenge and leads to undesirable outcomes, such as prolonged healing time and increased risk of non-union [6]. Bone defect resulting from non-union is a complex condition and the traditional treatment would be autologous bone grafts [7]. Nevertheless, autologous bone grafts do have significant disadvantages, such as the potential for postoperative and perioperative morbidity, the risks associated with prolonged general anaesthesia, the potential for surgical site infection due to increased surgical time, the limited availability of graft material, and the high cost of harvest [8,9,10]. Due to concerns regarding autografts, the use of synthetic bone grafts, especially the macroporous scaffold, has been studied in recent years.

Low-temperature rapid prototyping (LT-RP) is an emerging technique in 3D printing technology that employs polymeric solutions to fuse deposition and model scaffolds under −30 °C [11,12]. In previous reports, a poly (lactic-co-glycolic acid) (PLGA)/β-calcium phosphate (β-TCP) composite scaffold was established using the LT-RP technique and employed in challenging bone disease and defects [13,14]. The PLGA/TCP (P/T) scaffold was shown to be porous with interconnected macro- (492.8 ± 24.0 µm) and micropores (2–15 µm) [15]. Moreover, the P/T scaffold provided a stable biodegradation rate, more detail, the volume of the scaffold was decreased by 30% at week 4 and 80% at week 12 in vitro, as suggested by the previous study [16]. Furthermore, the slow release profile of encapsulating bioactive factors, excellent biocompatibility, and mechanical support in vivo, all make it an ideal biomaterial for the treatment of critical bone defects and steroid-associated osteonecrosis (SAON) [13,14,15,17,18].

Incorporating multiple osteogenic biomolecules such as growth factors, metal ions and drugs into an engineered biomaterial is a promising strategy to facilitate bone regeneration [14,19]. From this perspective, it is important to examine possible combinations among bioactive factors to maximize the additive-promoting effect on osteogenesis. Icaritin (ICT) is an intestinal metabolite derived from Chinese medicine Yin Yang Huo [20]. ICT has been developed for postmenopausal osteoporosis treatment due to its bone resorption inhibitory and bone formation enhancing effect [21]. Our previous study indicated that icaritin significantly promoted osteogenic differentiation and inhibited adipogenic differentiation of human MSCs [22]. Additionally, further studies revealed that sustained release of ICT has positive effects on bone defect healing when incorporated into P/T scaffolds [17,18]. Therefore, ICT possesses the potential for the treatment of osteoporosis and the healing of osteoporotic bone defects. On the other hand, secretome is defined as a set of secreted membrane-enclosed vesicles containing free nucleic acids and soluble proteins [23]. According to our previous studies, secretome derived from human fetal MSCs (HFS) ameliorated senescence in human adult MSCs, promoted osteogenic differentiation of adult MSCs, and enhanced bone consolidation in the distraction osteogenesis (DO) model, indicating its potential for stimulating bone regeneration in osteoporotic bones [24,25]. However, whether a combination of the natural ingredient (ICT) and the biological product (HFS) could additively accelerate osteoporotic bone healing remains to be determined.

**Scheme 1:**
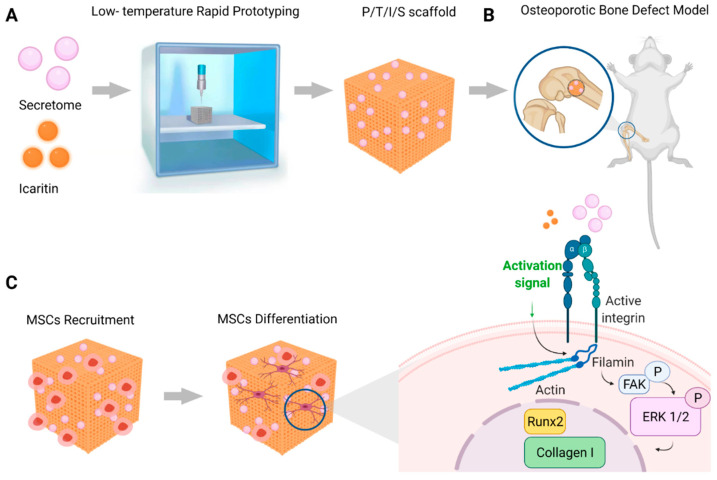
Schematic illustration of the fabrication of the bioactive porous scaffold and its application in osteoporotic bone regeneration. (**A**) The preparation of the P/T/I/S scaffold. (**B**) The application of the scaffold in osteoporotic bone defect healing. (**C**) The proposed underlying mechanism.

In the present study, we hypothesize that the combination of ICT and HFS could facilitate the bioactive property of PLGA/TCP-based porous scaffold, and thus provide a microenvironment favouring recruitment and differentiation of endogenous MSCs into osteoblast lineage to guide the osteoporotic bone regeneration (Scheme 1).

## 2. Materials and Methods

### 2.1. Production and Characterisation of HFS

A conditioned medium of human fetal MSCs was collected after primary culture and then subjected to concentration by using an Amicon Ultra-15 Centrifugal Filter Device (Millipore, Burlington, MA, USA), as in our previous report [24,26]. The concentrated supernatant was addressed as human fetal MSCs secretome (HFS).

For the quantification, the total protein concentration of the HFS was quantified by Pierce™ BCA Protein Assay Kit (Thermo Fisher Scientific, Waltham, MA, USA) at 562 nm according to the manufacturer’s manual.

Dynamic light scattering (DLS) analysis was carried out using the DelsaMax PRO Light Scattering Analyzer (Beckman Coulter, Brea, CA, USA) to measure the HFS size distribution. Detection of exosome marker (TSG101) from HFS by Western blot was performed as described in Section 2.6.

### 2.2. Rat MSCs Proliferation Study

MTS assay was performed to analyse the proliferative capacity of rat MSCs. Cells were trypsinized and seeded on 96-well plates (Corning, Kaiserslautern, Germany). At the indicated timepoint, 20 μL of MTS/PMS reagent (Promega, Madison, WI, USA) was added to each well and incubated at 37 °C for 4 h. The plate was read at 490 absorbance and the results were calculated.

### 2.3. Osteogenic Differentiation

MSCs were isolated from Sprague Dawley^®^ female rat bone marrow as previously described [27] and the rat MSCs (rMSCs) from passage 2 were employed in these experiments. For osteogenic induction, at about 80% confluence, rMSCs seeded in a 12-well plate were cultured with osteogenic induction medium (OIM), which is α-modified Eagle’s medium (αMEM) containing 10% fetal bovine serum (FBS), 1% penicillin/streptomycin, and supplemented with 10 nM dexamethasone, 50 μM ascorbic acid, and 10 mM β-glycerolphosphate (αMEM and FBS are from Gibco, Waltham, MA, USA; others are from Sigma-Aldrich, Burlington, MA, USA), in the presence of ICT (1 µM), HFS (50 µg/mL), and ICT (1 µM) combined with HFS (50 µg/mL), respectively. All the plates were incubated at 37 °C, 5% CO_2_.

At the indicated timepoint, the cell layers were washed with PBS twice and fixed with 70% ethanol for 10 min. The cells were incubated with Alkaline phosphatase (ALP) substrate solution (l mL ALP buffer with 5 μL BCIP, and 10 μL NBT, all from Promega, Madison, WI, USA) at 37 °C in the dark for 60 min. After that, plates were washed and air dried for imaging. For mineral bone nodule determination, Alizarin red staining (ARS) was performed [28]. Briefly, cell monolayers were washed with PBS twice and fixed with 70% ethanol for 10 min; after that, cells were stained by Alizarin red for 5 min followed by three washes with 50% ethanol.

### 2.4. RNA Extraction and Real-Time PCR

Cellular samples were subjected to RNA extraction at indicated timepoints, followed by Real Time-PCR(RT-PCR). Total cellular RNA was extracted with RNAiso Plus reagent and reversely transcribed into cDNA using PrimeScript RT Master Mix following the manufacturer’s protocol (all from TaKaRa, Kusatsu, Japan). The RT-PCR was performed by Power SYBR^®^ Green PCR Master Mix (Applied Biosystem, Waltham, MA, USA) with QuantStudio™ 7 Flex Real-Time PCR System (Thermo Fisher Scientific, Waltham, MA, USA). The relative fold changes of candidate genes were analysed using the 2^–ΔΔ^CT method normalized to the gene expression of *GAPDH*. The primer sequences used are listed in Table S1 (Supporting Information).

### 2.5. Cell Spreading

The rMSCs were trypsinized and suspended in αMEM without FBS, and then seeded on 14 mm diameter cover glasses (NEST^®^, Wuxi, China) in a density of 5 × 10^4^ cells/mL. After overnight fasting, cells were treated with or without ICT(1 µM), HFS (50 µg/mL), and ICT (1 µM) combined with HFS (50 µg/mL) for 1 day. Samples were washed by PBS followed by 4% paraformaldehyde fixation. After that, fixed samples were washed with PBS three times and then incubated with rhodamine-phalloidin (1:200, Cytoskeleton, Denver, CO, USA) at room temperature for 20 min. Samples were then mounted by ProLong™ Gold Antifade Mountant with DAPI (Thermo Fisher Scientific, Waltham, MA, USA). Cells were visualized under Leica DM5500 microscopy (Leica, Wetzlar, Germany).

### 2.6. Western Blot

After overnight fasting, rMSCs were treated with or without ICT (1 µM), HFS (50 µg/mL), and ICT (1 µM) combined with HFS (50 µg/mL) for 1 day. Total proteins of cultured cells were lysed by using radioimmunoprecipitation assay buffer (RIPA, from Sigma-Aldrich, Germany), supplemented with protease inhibitor cocktail and phosphatase inhibitor (all from Solarbio^®^ life sciences, Beijing, China). Cell lysates were centrifuged at 14,000 rpm at 4 °C for 10 min. Supernatants were then collected and mixed with sample loading buffer (Thermofisher, Waltham, MA, USA) prior to protein denature. Protein expressions were determined by Western blot analysis. Briefly, the boiled samples were loaded to SDS-PAGE gel and electrophoresed at a constant voltage of 100 V for 100 min. After that, the proteins from SDS-PAGE gel were transferred onto PVDF membranes at 100 V for 120 min. The membranes were then blocked with 5% bovine serum albumin (BSA) and probed with the following antibodies: integrin α3, Phospho-FAK, FAK, Phospho-Erk1/2, Erk1/2, and GAPDH. The information on antibodies used is listed in Table S2 (Supporting Information). The results were detected on the GeneGnome XRQ (Syngene, Cambridge, UK).

### 2.7. P/T and P/T/I Scaffold Fabrication

The porous scaffolds were fabricated using an LT-RP technique with a rapid prototyping-based fine spinning machine (CLRF-2000-ll, Tsinghua University, Beijing, China) [11]. For the preparation of PLGA/TCP (P/T) scaffolds, the PLGA (RESOMER; Boehringer-Ingelheim, Ingelheim, Germany) and β-TCP powders (Sigma-Aldrich, St. Louis, MA, USA) were dissolved in 1,4-dioxane (Sigma-Aldrich) at a 1:4 *w*/*w* ratio to form a homogeneous paste for printing, as previously described [16]. For the preparation of PLGA/TCP/icaritin (P/T/I) scaffolds, icaritin (ICT, Yuanye Biotechnology, Shanghai, China) was dissolved in 1,4-dioxane thoroughly, then added into the PLGA/TCP mixture at a ratio of 160 mg ICT: 100 g PLGA/TCP, and stirred vigorously to form a homogeneous paste for printing [29]. After that, the pastes were employed to print a cubic scaffold (20 mm in length) with a 500 µm default pore size under −28 °C. Finally, the scaffolds were lyophilized in a freeze dryer for 24 h (Bo Yi Kang FD-1-50, Beijing, China) under a vacuum of 20–40 Pa pressure.

### 2.8. Preparation of P/T/I Scaffold Loading with HFS and HFS Labelling

The P/T or P/T/I scaffolds were trimmed into desired cylinders (size of 2 mm in diameter and 4 mm in length) and then immersed in HFS solution in glassware with a vacuum valve, and then the air within the scaffolds was removed by vacuum followed by the HFS solution penetration. The amount of HFS loaded into the scaffold was 466.7 ± 9.4 µg protein per cylinder. HFS were labelled with PKH26 Red Fluorescent Cell Linker Midi Kit (Sigma-Aldrich, Burlington, MA, USA) according to the manufacturer’s protocol. More specifically, PKH26 dye was diluted in Diluent C containing HFS solution. Excess dye was quenched by 10% BSA (Sigma, Burlington, MA, USA) in PBS. After adding 0.971 M sucrose solution, the solution was centrifuged at 190,000× *g* for 2 h at 4 °C. The pellet was resuspended and transferred to Amicon 10 kDa MWCO (Millipore, Burlington, MA, USA), and then spun at 3000× *g* for 40 min. The concentrate from the Amicon column was PKH26-labelled HFS and showed red fluorescence (ex/em ~551 nm/~567 nm).

### 2.9. Determination of HFS Released from PLGA/TCP In Vitro

During the in vitro degradation process, the scaffold was dipped in saline as the degradation medium and was kept for over two weeks. The scaffold was placed in the test tube, along with ten times its volume of saline. The saline was collected and replenished at 1 h, 4 h, 6 h, 1 day, 2 days, 4 days, 7 days, and 14 days. The total protein concentration of the HFS was quantified by Pierce™ BCA Protein Assay Kit (Thermo Fisher Scientific, Waltham, MA, USA) at 562 nm, according to the manufacturer’s manual. 

### 2.10. Animal Study

Mature SD female rats (16 weeks old, mean body weight of 250 g) were provided by the Laboratory Animal Research Centre of the Chinese University of Hong Kong. For surgery, the animals had general anaesthesia induced by intraperitoneal injection of xylazine (2.5 mg/kg) and ketamine (50 mg/kg) in saline. OVX was performed as previously described [30]. Briefly, the bilateral ovaries of rats were removed through a dorsal approach. Twelve weeks after an OVX model was established, cylindrical defects (2 mm) were created through the distal femur of OVX rats, laying above the growth plate, as described earlier [31]. After that, the defect sites were implanted with or without different scaffolds (2 mm in diameter and 4 mm in length). Animals were randomly selected for termination at 4 weeks, 8 weeks, and 12 weeks post-implantation. The bone-defected rats were divided into five groups as shown below. CON: osteoporotic bone defect rats without implantation, P/T: osteoporotic bone defect rats implanted with the PLGA/TCP scaffold, P/T/I: osteoporotic bone defect rats implanted with the PLGA/TCP/ICT scaffold, P/T/S: osteoporotic bone defect rats implanted with the PLGA/TCP scaffold loaded with HFS, P/T/I/S: osteoporotic bone defect rats implanted with PLGA/TCP/ICT loaded with HFS (*n* = 6 per group per timepoint).

### 2.11. Micro-CT Analysis

The microstructure of distal femoral metaphysis was analysed using high-resolution vivaCT 40 (Scanco Medical, Bruttisellen, Switzerland), as previously reported [32,33]. Image acquisition was performed at 70 kV and 114 μA, with a resolution of 17.5 μm per voxel, and the segmentation parameters for the bone from the background were fixed at Sigma = 0.8, Support = 2, and Threshold = 158–1000 [34]. For the distal femur 3D reconstruction, the VOI was 530 slices starting from the top. As for the bone defect 3D reconstruction, a consistent region of interest (ROI) was located in the central 1 mm diameter circle of the defected site, and the VOI of this region was 160 slices. Bone volume/tissue volume (BV/TV), and bone mineral density (BMD) were determined with a built-in program (Image Processing Language v4.29d, Scanco Medical, Bruttisellen, Switzerland).

### 2.12. Histology and IHC

All femurs were fixed with 4% PFA at 4 °C for 48 h, followed by decalcification in 10% ethylenediaminetetraacetic acid (EDTA) solution for 4 weeks. After that, decalcified samples were subjected to paraffin embedding and then sliced into 5 µm sections by a rotary microtome (HM 355S; Thermo Fisher Scientific, Waltham, MA, USA).

For bone morphology evaluation, Goldner’s trichrome staining was performed as described in the previous study [35]. Immunohistomorphology (IHC) was performed according to a standard protocol as previously described [36]. After deparaffinization, sections were treated with primary antibodies to Vimentin, SRY-Box Transcription Factor 2 (Sox2), Runx2, and Collagen Ⅰ, and a horseradish peroxidase-streptavidin detection system (Dako, Santa Clara, CA, USA) was used, followed by counterstaining with haematoxylin. Images were taken under Leica DM5500 microscopy (Leica, Wetzlar, Germany). The overview images of the defect site from the specimen were analysed (*n* = 4 per group). Semi-quantitative analysis of IHC was performed using the IHC profiler plugin, ImageJ (NIH, Bethesda, MD, USA). IHC profiler separated the DAB colour spectra by using optimised optical density vectors of the colour deconvolution plugin. After that, pixel-by-pixel analysis of the DAB-stained image was performed, followed by the display of the full profile along with its scoring decision. It is possible to use this tool globally to score most protein targets where the marker protein expression is cytoplasmic or nuclear in nature [37]. The information on used antibodies is listed in Table S2 (Supporting Information).

### 2.13. Statistical Analysis

All experiments were carried out in triplicate and the results were presented as mean ± SD and analysed using GraphPad PRISM^®^ (GraphPad Software, San Diego, CA, USA. One-way ANOVA was used for comparisons and Tukey’s HSD was used for the post hoc test. Statistical analysis was performed with SPSS (Version 20); a *p*-value < 0.05 was considered statistically significant.

## 3. Results

### 3.1. The Additive Effect of ICT and HFS on the Osteogenesis of rMSCs

Firstly, the effect of ICT and HFS on rMSCs proliferation was studied by MTS assay. Results showed that ICT concentrations ranging from 0.78125 to 12.5 µM did not cause a significant difference in cell proliferation over a 7-day period when compared to the control group. Nonetheless, on day 7, ICT concentrations greater than 25 µm significantly inhibited cell proliferation. On the other hand, HFS significantly promoted cell proliferation activity in the range between 0 and 200 µg/mL for 7 days of culture, while this promoting effect was reversed when 400 µg/mL of HFS or above was added (Figure S3, supporting information).

**Figure 1:**
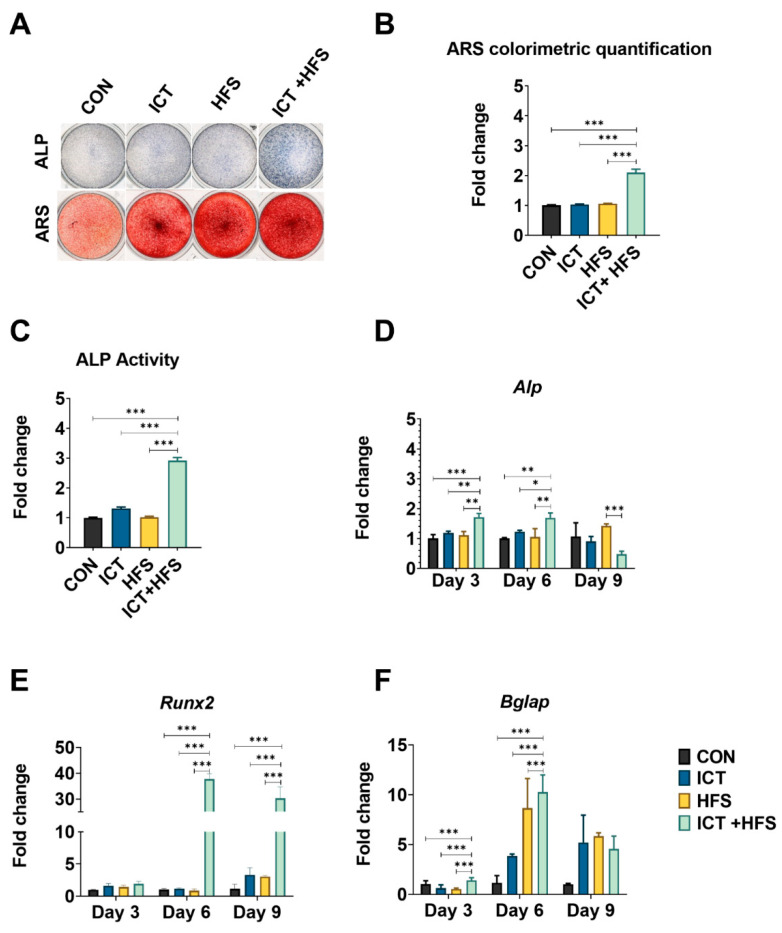
The additive effect of ICT and HFS on osteogenic differentiation of rMSCs. Cells were treated with ICT (1 µM), HFS (50 µg/mL), or their combination (ICT (1 µM) + HFS (50 µg/mL)). The cells treated with the vehicle served as controls (CON). (**A**) Top: ALP activity. Bottom: ARS staining. (**B**,**C**) Semi-quantitative analysis of ALP and ARS staining. (**D**–**F**) mRNA expression levels of osteogenic markers *Alp*, *Runx2*, and *Bglap*. Data are shown as mean ± SD (* *p* < 0.05, ** *p* < 0.01, *** *p* < 0.001, one-way ANOVA with Tukey’s HSD, *n* = 3).

Next, the osteogenic effect of ICT combined with HFS on rMSCs was evaluated. ALP is highly expressed in differentiated osteoblasts, and ALP activity is normally used as a marker of osteoblast activity [38]. Results showed that ALP was more active in the combination (ICT and HFS) group, being almost three-fold higher than that in the control, ICT, and HFS groups. The mineral deposition is a feature of late-stage osteogenic differentiation which can be evaluated by ARS. In the combination group, the number of mineral bone nodules was two times higher than that of the other groups on day 7 after osteogenic induction (Figure 1A–C).

To further illustrate the enhanced osteogenesis of rMSCs by ICT and HFS, three osteogenic markers, including *Alp*, *Runx2*, and bone gamma-carboxyglutamate protein (*Bglap*) [39] were studied during the osteogenic induction of rMSCs (Figure 1D–F). According to the RT-PCR results, the combination of ICT and HFS significantly increased the mRNA expression level of *Alp* on days 3 and 6, and *Runx2* expression on days 6 and 9 of osteogenesis induction in comparison to the other three groups. Compared to the control, ICT, and HFS group, *Bglap* expression was significantly increased on days 3 and 6 of the osteogenic induction process in the combination group. In summary, ICT and HFS were found to additively promote the osteogenesis of rMSCs. This suggests that the combination possesses the potential to enhance the biomineralization ability of the P/T scaffold.

### 3.2. ICT and HFS Additively Promote Osteogenic Differentiation of rMSCs through Integrin-mediated Focal Adhesion Pathway

**Figure 2:**
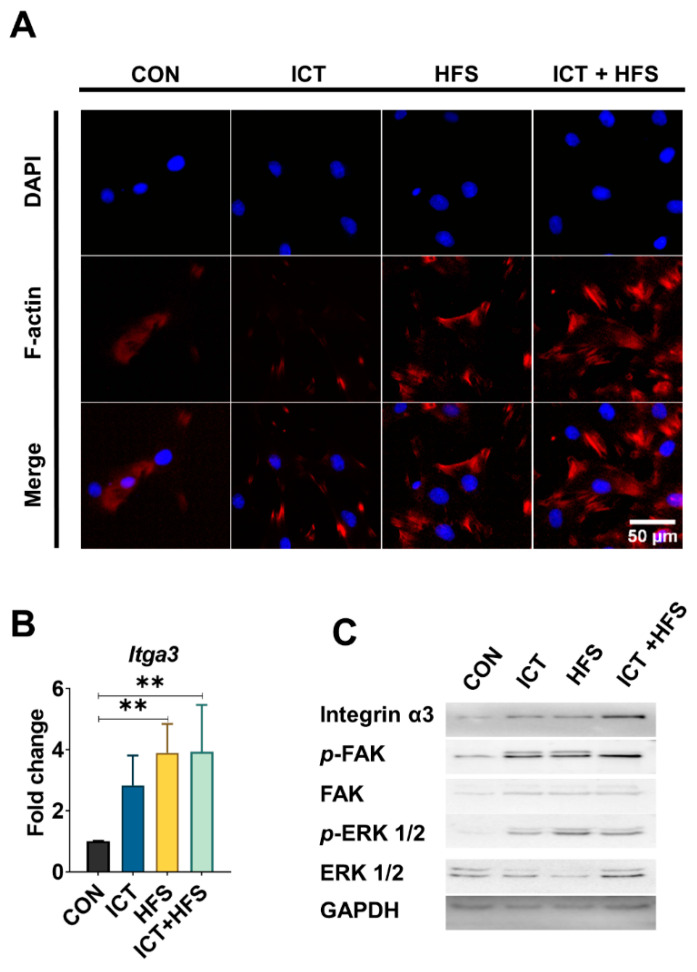
The additive effect of ICT and HFS on integrin-mediated focal adhesion pathway. Cells were treated with ICT (1 µM), HFS (50 µg/mL), or their combination (ICT (1 µM) + HFS (50 µg/mL)). The cells treated with the vehicle served as controls (CON). (**A**) Representative images of rMSCs spreading. F-actin and cell nuclei were labelled with red and blue fluorescent, respectively. (**B**) mRNA levels of *Itga3*. Data are shown as mean ± SD (** *p* < 0.01, one-way ANOVA with Tukey’s HSD, *n* = 3). (**C**) The effect of the combination of ICT and HFS on integrin subunits and intercellular signalling evaluated by Western Blot.

As the binding of integrins to matrix proteins governs the cell skeletal dynamics and cellular structure [40], phalloidin staining of the actin filament (F-actin) was performed. In the combination group, early cell spreading could be found by well-distributed F-actin (Figure 2A).

To further determine which key integrin was targeted by the combination of ICT and HFS, mRNA expression of integrin subunits in rMSCs after treatment was measured. As shown in Figure 2B and Figure S4, the ICT and HFS combination significantly upregulated the gene expression of integrin subunit β1 (*Itgb1*) by 2-fold (*p* < 0.01) and integrin subunit α3 (*Itga3*) expression, four-fold (*p* < 0.01) compared to the control group. Specifically, the protein expression of integrin α3 was significantly upregulated after the combination treatment compared to the control group (Figure 2C). As shown in the results (Figure 2C), FAK and ERK 1/2 proteins were significantly activated by ICT and HFS treatment in comparison with the other three groups.

### 3.3. Bioactive Scaffold Maintains Sustained Release of Bioactive Factors and Supports the Proliferation of Cells

**Figure 3:**
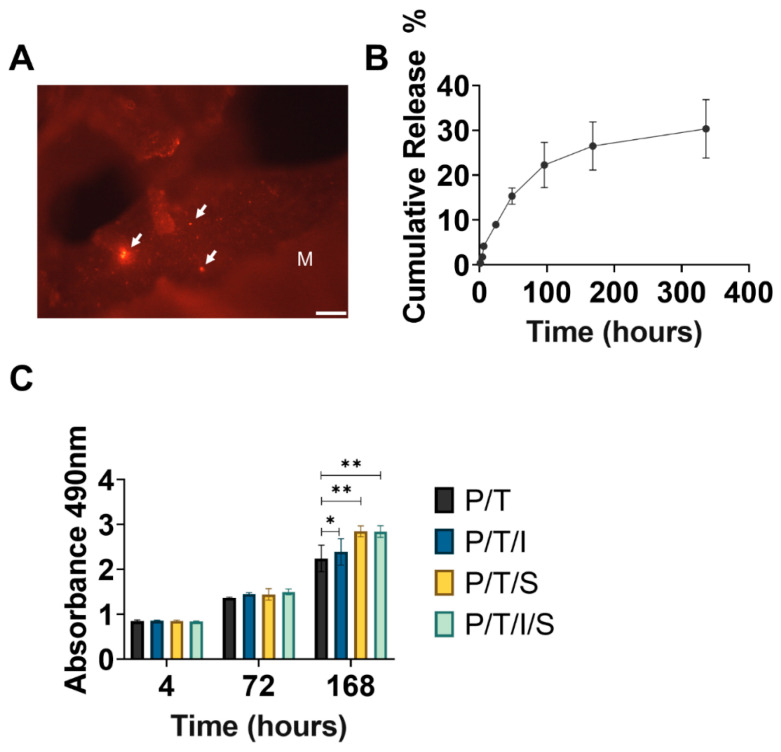
Loading and release of HFS in the P/T-based scaffolds. (**A**) P/T/I scaffold incorporating HFS (466.7 ± 9.4 µg protein per cylinder). White arrows indicate PKH26-stained extracellular vesicles of HFS, scale bar = 20 µm, M:material. (**B**) Kinetics of HFS released from the P/T/I/S scaffold in vitro. (**C**) Cell proliferation. Data are shown as mean ± SD (* *p* < 0.05, ** *p* < 0.01, one-way ANOVA with Tukey’s HSD, *n* = 3).

To incorporate ICT and HFS into the P/T scaffold, an ICT solution was added to the P/T bioink in the 3D printing process. After that, HFS was absorbed into the P/T/I scaffold by vacuum to generate a bioactive scaffold. Figure 3A indicates that PKH26-labelled HFS was successfully loaded into the scaffold. In addition, the release kinetics showed that HFS could be sustainably released from the P/T/I/S scaffold for up to 14 days (Figure 3B). Our previous study has shown that ICT was gradually released from the P/T/I scaffold in 12 weeks [15].

Therefore, these findings suggested that the P/T scaffold can serve as a promising delivery system for the sustained release of ICT (a hydrophobic small molecule) and HFS (biological products). Next, the in vivo effect of P/T/I/S was investigated.

### 3.4. Bioactive P/T/I/S Scaffold Enhances Osteoporotic Bone Regeneration

**Figure 4:**
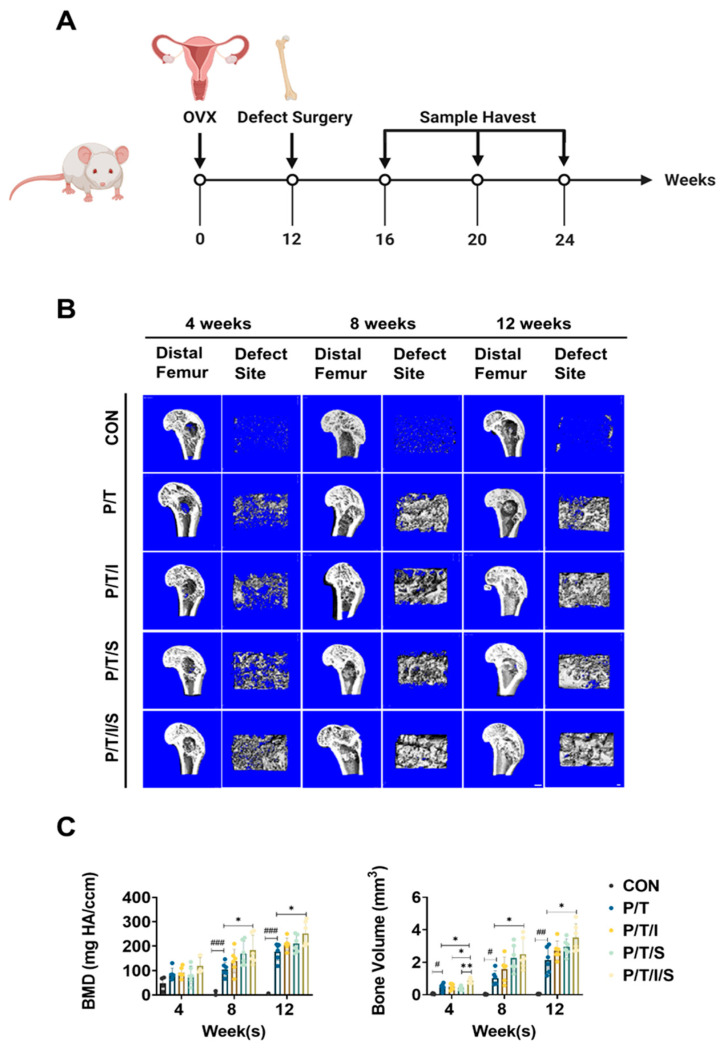
Osteoporotic bone regeneration in distal femoral defect. (**A**) The timeline of animal surgery. (**B**) Three-dimensional reconstructed mineralized tissue at defect sites of distal femurs at 4, 8, and 12 weeks after implantation. The scanning resolution is 17.5 µm at 70 kV,114 µA and the threshold is 158–1000. (**C**) Quantitative data of micro-CT. Data are shown as mean ± SD (# *p* < 0.05, ## *p* < 0.01, ### *p* < 0.001, denotes significant differences between the control and P/T group. * *p* < 0.05, ** *p* < 0.01, denotes significant differences among P/T, P/T/I, P/T/S, and P/T/I/S groups, one-way ANOVA with Tukey’s HSD, *n* = 6).

To promote osteoporotic bone defect healing, we innovatively applied the P/T/I/S scaffold to a bone defect in an osteoporotic rat model. After induction of osteoporosis by OVX, P/T, P/T/I, P/T/S, and P/T/I/S scaffolds were implanted into the bone defects of distal femurs in OVX rats (Figure 4A). Micro-CT analysis and histomorphology were employed to evaluate bone regeneration. According to the 3D reconstruction result (Figure 4B), newly formed bone was rarely found at the defect site in the control group even 12 weeks following defect surgery, suggesting the healing of osteoporotic bone defects is challenging without implantation. At week 4 post-implantation, all the groups with implantation achieved bone formation to various degrees. An abundance of woven bone was especially evident in the P/T/I/S group. At 8 weeks after defect surgery, the regenerated bone had already mineralized in the P/T/I, P/T/S, and P/T/I/S groups, while the P/T group still showed un-mineralized tissue at the defect site (Figure 4B). At week 12, the mineralized bone integrated with the host bone in the P/T/I, P/T/S, and P/T/I/S groups, but not in the P/T group.

Furthermore, BV and BMD were calculated to determine bone mass at the defect sites. Both BV and BMD were increased in a time-dependent manner in all the groups with implantations. Moreover, the P/T/I/S group was superior to other groups for the duration of the study. For example, the BV in the P/T/I/S group was 0.84 ± 0.15 mm^3^, which was 2 times that of the P/T/S (0.41 ± 0.11 mm^3^, *p* < 0.01) and 1.68 times that of the P/T/I group (0.50 ± 0.18 mm^3^, *p* < 0.05) at 4 weeks after implantation. Furthermore, the BV and BMD were also significantly higher in the P/T/I/S group at week 8 and week 12, compared to the control group (Figure 4C).

**Figure 5:**
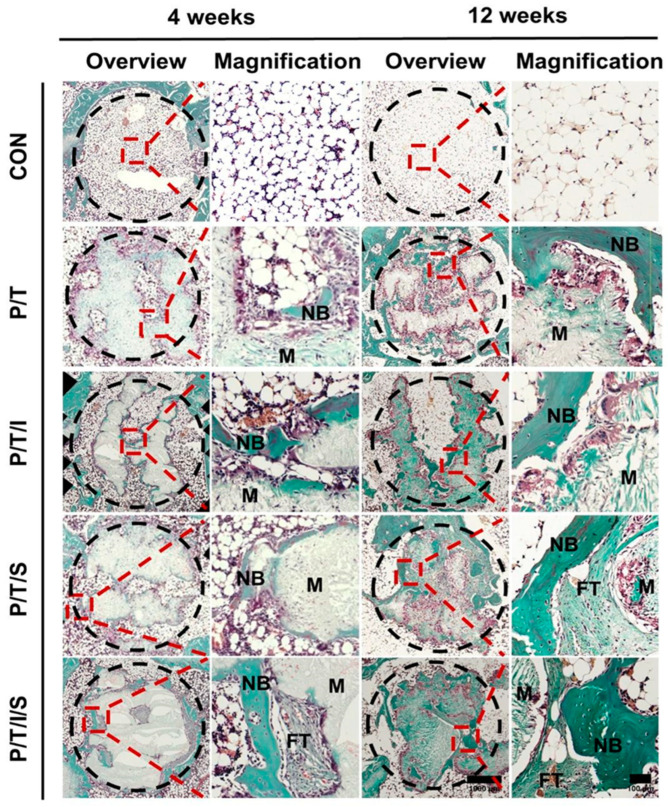
Representative histological images of femoral bone defect at 4 or 12 weeks. Samples were stained by Goldner’s trichrome. The black dashed circle indicates the defect site, and the red dash rectangle indicates the magnified area. NB: new bone, FT: fibrous tissue, M: scaffold materials. For overview images, the scale bar is 1000 µm, while for magnification images it is 100 µm.

Histological analysis was performed by Goldner’s trichrome staining (Figure 5). In the control group, bone-like tissue was seldom present but lots of adipocytes could be found at the defect site. On the contrary, a large amount of new bone tissue (green colour) was found in and around the implant in the P/T/I/S group at week 12, which was consistent with the micro-CT reconstruction result. In the P/T group, there was collagen (red colour) around the implant rather than mineralized bone, indicating that the connective tissue had not yet undergone mineralization.

In summary, our results from the animal study indicate that the P/T scaffold incorporating ICT and HFS enhances the regenerative capacity of osteoporotic bone by significantly promoting bone regeneration at the defect sites.

### 3.5. Bioactive P/T/I/S Scaffold Recruits Endogenous MSCs to Boost the Healing of Osteoporotic Bone

**Figure 6:**
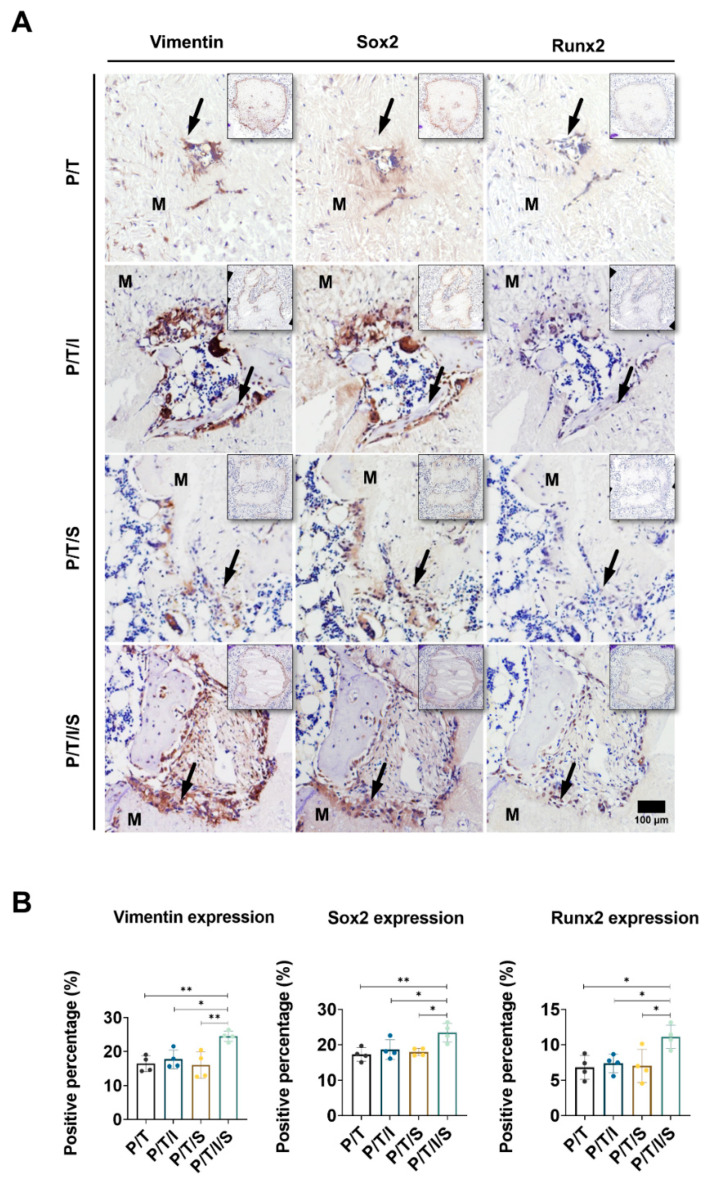
Expression of cellular adhesive, pluripotent, and osteogenic markers in femoral bone defect at week 4. (**A**) IHC staining for Vimentin, Sox2, and Runx2 on adjacent sections 5 μm apart. Black arrows indicate the positive signal of the target protein. M: materials. (**B**) Semi-quantitative analysis of Vimentin, Sox2, and Runx2 expression at defect sites shown in (**A**). Data are shown as mean ± SD (* *p* < 0.05, ** *p* < 0.01, one-way ANOVA with Tukey’s HSD, *n* = 4).

To evaluate cell recruitment and cell differentiation in vivo, IHC was performed on consecutive slides (Figure 6). Vimentin is an intermediate filament protein that is expressed in MSCs and governs cell migration [41,42], while Sox2 is a representative marker of pluripotent cells and has a functional role in fracture healing [43,44]. To provide a detailed characterization of the cellular phenotype at the defect site, we analysed the spatial expression of Vimentin and Sox2. According to the IHC staining result, we found that endogenous MSCs with pluripotent were recruited to the scaffolds. Notably, the expressions of Vimentin and Sox2 (25.54 ± 1.33% and 23.4 ± 2.31%, respectively) were significantly upregulated in the P/T/I/S group compared to the control group (16.42 ± 2%, *p* < 0.01 and 17.22 ± 1.66%, *p* < 0.01, respectively).

Runx2 is a master control gene of osteogenic differentiation [45], and collagen I is the major component of the bone matrix [39]. According to the results, a large number of recruited cells in the P/T/I/S group exhibited intense positive staining against Runx2 and collagen I (Figure 6 and Figure S6), which is consistent with the in vitro findings. In more detail, compared to the control group (6.79 ± 1.45%), the expression of Runx2 increased markedly in the P/T/I/S group (11.11 ± 1.44%, *p* < 0.05).

## 4. Discussion

The characteristics of the P/T scaffold, including interconnected macropore and degradation rate, facilitated new vessels and bone ingrowth [15]. However, the P/T scaffold presented a restricted efficiency in bone regeneration due to its limited bioactivity [13]. In order to improve the bone regeneration efficiency of the P/T scaffold, great efforts have been made to regulate its bioactive property [13,14]. A previous study reported that an efficient way to evaluate the osteogenic potential of the P/T scaffold is by ICT incorporation [29]. The efficacy of the P/T/I scaffold was tested on a bone defect rabbit model and the bone volume was increased by the P/T/I scaffold compared to that of the P/T group. However, the underlying mechanism has not been fully explored.

In this study, we found an additive effect of ICT and HFS on the osteogenesis of rMSCs in vitro, supporting the rationale of combining ICT and HFS in promoting bone defects in an animal model by the existing PLGA/TCP scaffold. Based on the results of the animal study, we also showed an additive effect of ICT and HFS in promoting bone regeneration in the osteoporotic defect model, suggesting consistent results with in vitro studies. According to Micro-CT and histological results, the P/T/I/S groups showed superior function in promoting new bone regeneration in the osteoporotic bone to the P/T, P/T/I, and P/T/S groups. Overall, the incorporation of ICT and HFS enhanced the bone formation performance of the P/T scaffold.

Although we did not show the cell attachment on the scaffolds in the cellular study, we straightforwardly demonstrated that the cell-free scaffold recruited MSCs in vivo. We also elucidated that the P/T/I/S scaffold increased the expression of cell adhesive (Vimentin), MSCs pluripotent (Sox2), and osteogenic markers (Runx2 and collagen I) at the defect site. In summary, we demonstrated how the combination of ICT and HFS benefits the bioactive potential of the P/T scaffold. Firstly, the P/T/I/S scaffold effectively promoted endogenous MSCs homing to the defect site; secondly, the pluripotency of rMSC in the osteoporotic rat could be restored by ICT and HFS released from the P/TI/S scaffold implantation. The recruited MSCs could be further differentiated into osteoblast, and eventually, the P/T/I/S scaffold remarkably enhanced the capability of osteoporotic bone regeneration. The bioactive enhancement of the P/T scaffold could be associated with the activation of the Integrin–FAK–ERK1/2–Runx2 axis by the incorporation of ICT and HFS.

Integrins are a family of cell transmembrane proteins consisting of α chains and β chains, allowing the homing of MSCs to bone and the attachment of osteoblast and osteoclast to the extracellular matrix (ECM) [46,47]. The binding of integrins to ECM triggers integrin clustering, activates the intracellular signalling proteins such as FAK, and subsequently leads to the phosphorylation of ERK1/2, phosphatidylinositol 3-kinase (PI3K), RACα serine/threonine-protein kinase (Akt), and others [47,48,49]. In particular, the activation of FAK–ERK1/2 promotes osteogenic differentiation in MSCs [47]. Moreover, it has been reported that the Runx2 expression in MSCs was induced by the activation of the FAK–ERK 1/2 signalling pathway coupled with integrins [50,51].

In this study, we demonstrated that the combination of ICT and HFS treatment significantly increased the expression of integrin α3 in vitro. In addition, the combination effectively increased the FAK–ERK1/2 phosphorylation. Furthermore, the combination elevated the ALP activity and mineralization of MSCs, as well as increased the mRNA level of osteogenic markers such as *Alp*, *Runx2*, and *Bglap*. These results suggested that the combination of ICT and HFS contributes to bone regeneration by enhancing the recruitment and osteogenic differentiation of MSCs, which could be associated with the Integrin–FAK–ERK1/2–Runx2 axis. However, the interaction between α3 integrin and the combination of ICT and HFS has not been investigated in the study. In addition, further studies such as in vitro and in vivo blockage assays are needed to fully support the combination regulating bone formation through this axis.

## 5. Conclusions

With the sustained release of ICT and HFS from the P/T/I/S scaffold, the cell-free P/T/I/S scaffold efficiently promoted bone regeneration at the femoral defect site in the OVX-induced osteoporotic rat model, which could be related to an enhancement in recruitment and osteogenic differentiation of endogenous MSCs. Furthermore, the in vitro mechanism studies revealed that the combination of ICT and HFS additively promoted MSCs osteogenesis potential, which could be associated with the Integrin–FAK–ERK–Runx2 axis. This bioactive scaffold provides new insight useful for developing novel therapies for the argumentation of difficult bone healing conditions, such as osteoporotic fractures or defects.

## Data Availability

Not applicable.

## Supplementary Materials

The following supporting information can be downloaded at: https://www.mdpi.com/article/10.3390/bioengineering9100525/s1, Figure S1: Particle size distribution of secretome derived from human fetal mesenchymal stem cells (HFS); Figure S2: Western blotting of the exosome marker TSG101. A&B: different batch of HFS; Figure S3: Proliferation of the rat mesenchymal stem cells (rMSCs) treated with different concentrations of ICT (A) and HFS (B) for 7 days culture, respectively; Figure S4: mRNA levels of integrin subunits in rMSCs; Figure S5: Representative fluorescent images of PKH26-positive particles in PKH26-labeled HFS-free control (CON) and in PKH26-labeled HFS (HFS); Figure S6: Representative images of bone defect samples at 4 weeks post bone defect surgery stained by immunohistochemical staining for collagen Ι (COL-Ι); Table S1: Sequences of primers used for real-time PCR; Table S2: List of antibodies.
