# Supplementary material for: Bioactive Scaffold Fabricated by 3D Printing for Enhancing Osteoporotic Bone Regeneration
Source: Bioengineering (Basel). 2022 Oct 5;9(10):525. doi: 10.3390/bioengineering9100525 (PMC9598556; doi:10.3390/bioengineering9100525)
Supplement: Supplementary file 1 [file bioengineering-09-00525-s001.zip › bioengineering-1916306-supplementary.pdf]

bioengineering

MDPI

Supporting Information

Bioactive Scaffold Fabricated by 3D Printing for Enhancing Osteoporotic Bone Regeneration

Xiaoting Zhang [1,2,†], Xinluan Wang [3,†], Yuk-wai Lee [4,5,6], Lu Feng [1,2], Bin Wang [1,2], Qi Pan [1,2], Xiangbo Meng [3], Huijuan Cao [3], Linlong Li [1,2], Haixing Wang [1,2], Shanshan Bai [1,2], Lingchi Kong [1,2], Dick Ho Kiu Chow [1,2], Ling Qin [1,2], Liao Cui [7], Sien Lin [1,2,*] and Gang Li [1,2,*]

1 Musculoskeletal Research Laboratory, Department of Orthopaedics & Traumatology, The Chinese University of Hong Kong, Prince of Wales Hospital, Hong Kong SAR, China
2 Stem Cells and Regenerative Medicine Laboratory, Li Ka Shing Institute of Health Sciences, The Chinese University of Hong Kong, Prince of Wales Hospital, Hong Kong SAR, China
3 Shenzhen Institute of Advanced Technology, Chinese Academy of Sciences, Shenzhen 518055, China
4 SH Ho Scoliosis Research Laboratory, Department of Orthopaedics and Traumatology, The Chinese University of Hong Kong, Hong Kong SAR, China
5 Joint Scoliosis Research Center of the Chinese University of Hong Kong and Nanjing University, The Chinese University of Hong Kong, Hong Kong SAR, China
6 Li Ka Shing Institute of Health Sciences, The Chinese University of Hong Kong, Hong Kong SAR, China
7 School of Pharmacy and Guangdong Key Laboratory for Research and Development of Natural Drugs, Guangdong Medical University, Zhanjiang 524023, China
* Correspondence: sienlin@cuhk.edu.hk (S.L.); gangli@cuhk.edu.hk (G.L.)
† These authors contributed equally to this work.

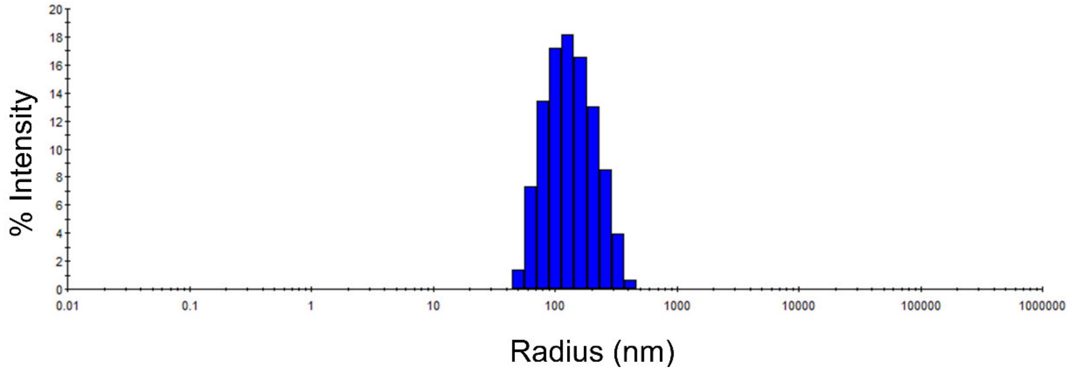

Figure S1. Particle size distribution of secretome derived from human fetal mesenchymal stem cells (HFS).

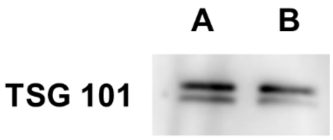

Figure S2. Western blotting of the exosome marker TSG101. A&B: different batch of HFS.

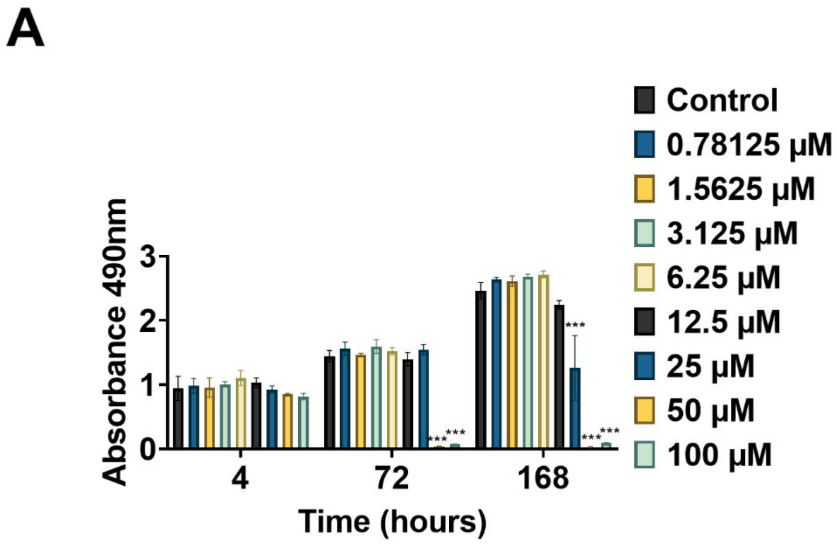

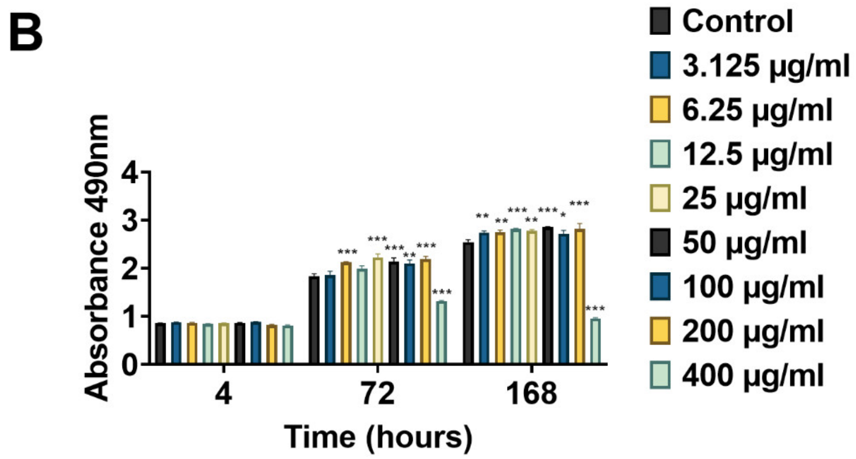

Figure S3. Proliferation of the rat mesenchymal stem cells (rMSCs) treated with different concentrations of ICT (A) and HFS (B) for 7 days culture, respectively. Data were shown as mean ± SD (*$P < 0.05$, **$P < 0.01$, ***$P < 0.001$, one-way ANOVA with Tukey's HSD, n=3).

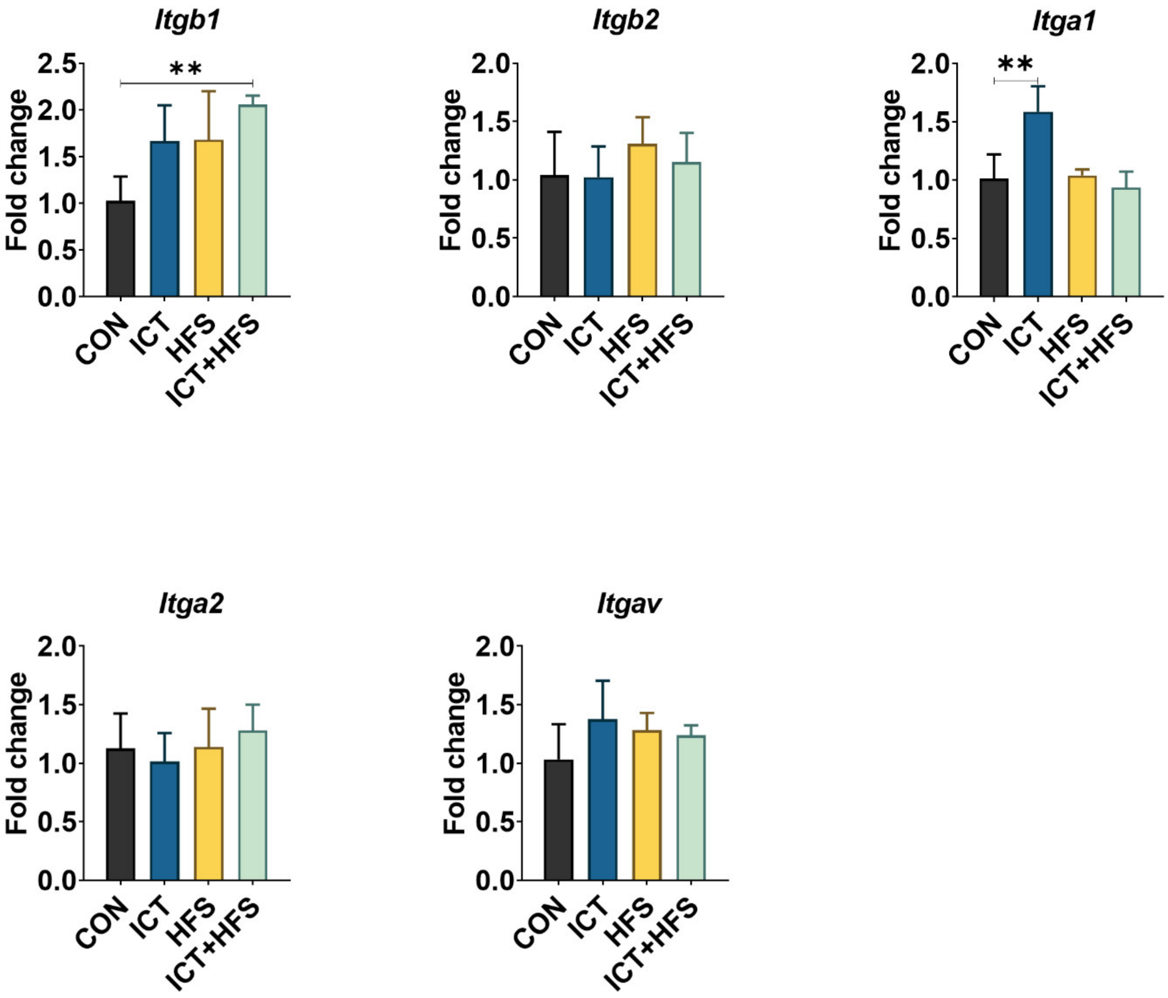

Figure S4. mRNA levels of integrin subunits in rMSCs. Data were shown as mean ± SD. (**P < 0.01, one-way ANOVA with Tukey's HSD, n=3).

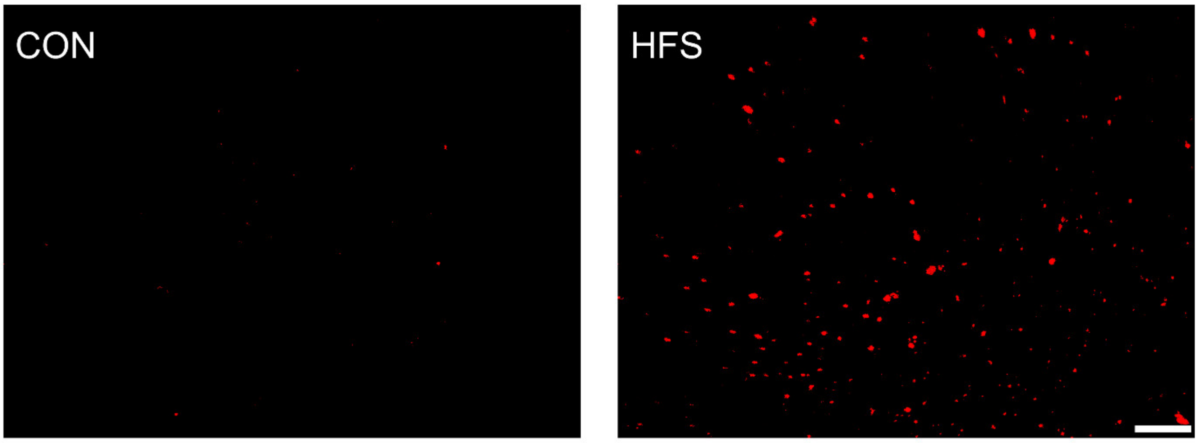

Figure S5. Representative fluorescent images of PKH26-positive particles in PKH26-labeled HFS-free control (CON) and in PKH26-labeled HFS (HFS). Scale bar = 2μm.

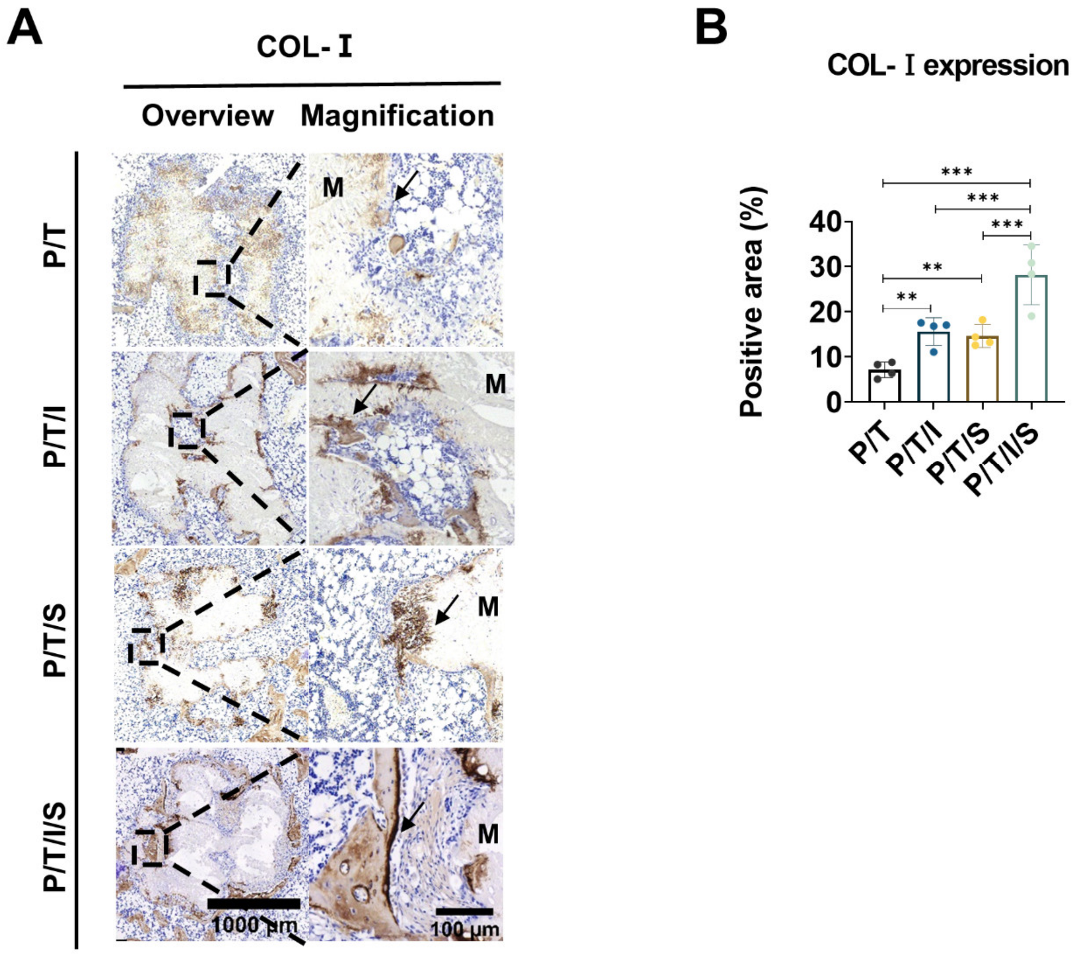

Figure S6. Representative images of bone defect samples at 4 weeks post bone defect surgery stained by immunohistochemical staining for collagen I (COL-I). M:materials, black arrows indicate the positive signal of target protein. B) Semi-quantitative analysis of new bone formation expressed COL-I levels at 4 weeks after the implantation. Data were shown as mean ± SD (*P < 0.05, **P < 0.01, ***P < 0.001, one-way ANOVA with Tukey's HSD, n=4.).

Table S1. Sequences of primers used for real-time PCR.

Gene	Forward	Reverse
Gapdh	5′AGCCCAGAACATCATCCCTG′3	5′CACCACCTTCTTGATGTCATC′3
Alp	5′CCTTAGGGCCACCGCTCG′3	5′GTTCAGTGCGGTTCCAGACA′3
Runx2	5′CCGATGGGACCGTGGTT′3	5′CAGCAGAGGCATTTCGTAGCT′3
Bglap	5′CTCAACAATGGACTTGGAGCC′3	5′GTCCGCTAGCTCGTCACAAT′3
Itgb1	5′CAAGTGGGACACGGGTGAA′3	5′AGCTTGATTCCAAGGGTCCG′3
Itgb2	5′AAGCCTGCAAGGAGGAACTC′3	5′CCACTGACGGGTAGTCGAAC′3
Itga1	5′AGTCCACGAACACATTCCCT′3	5′TGCTGGGACTTGACGATCAG′3
Itga2	5′ CACTGCCACCTGTGAAAAGC′3	5′ AGGACCGCAGGTTAGAAAGC′3
Itga3	5′CAAACGGAACCAGAGGATGG′3	5′TAGCTTCATACAGGGCACGA′3
Itgav	5′ TGGCACAAAGACCGTTGAGT′3	5′AGCTCCGACAACTAAGTCTGGA′3

Table S2. List of antibodies.

Product name	Catalog number	Supplier	Application
Integrin α3 Polyclonal Antibody	YT2362	Immunoway	Western Blot: 1/1000
Phospho-FAK (Tyr397) Antibody	#3283	Cell signaling technology	Western Blot: 1/1000
FAK Antibody	#3285	Cell signaling technology	Western Blot: 1/1000
Phospho-p44/42 MAPK (Erk1/2) (Thr202/Tyr204) (D13.14.4E) XP® Rabbit mAb	#8544	Cell signaling technology	Western Blot: 1/1000
p44/42 MAPK (Erk1/2) (137F5) Rabbit mAb	#4695	Cell signaling technology	Western Blot: 1/1000
GAPDH Antibody (FL-335)	sc-25778	Santa Cruz Biotechnology	Western Blot: 1/1000
Anti-RUNX2 antibody	ab76956	abcam	Immunohistochemistry: 1/200
Recombinant Anti-Vimentin antibody	ab92547	abcam	Immunohistochemistry: 1/500
Anti-SOX2 antibody	ab97959	abcam	Immunohistochemistry: 1/200
Recombinant Anti-TSG101 antibody	ab125011	abcam	Western Blot: 1/1000
